# Long-Term Soil Drought Limits Starch Accumulation by Altering Sucrose Transport and Starch Synthesis in Sweet Potato Tuberous Root

**DOI:** 10.3390/ijms24033053

**Published:** 2023-02-03

**Authors:** Minfei Sheng, Houqiang Xia, Huizi Ding, Dongyu Pan, Jinping He, Zongyun Li, Jingran Liu

**Affiliations:** 1 Institute of Integrative Plant Biology, School of Life Sciences, Jiangsu Normal University, Xuzhou 221116, China; 2 Jiangsu Key Laboratory of Phylogenomics and Comparative Genomics, School of Life Sciences, Jiangsu Normal University, Xuzhou 221116, China

**Keywords:** sweet potato, sucrose and starch metabolism, drought treatment, acid invertase, sucrose-will-eventually-be-exported transporter (*SWEET*)

## Abstract

In this study, the influences of long-term soil drought with three levels [soil-relative water content (*SRWC*) (75 ± 5)%, as the control; *SRWC* (55 ± 5)%, mild drought; *SRWC* (45 ± 5)%, severe drought] were investigated on sucrose-starch metabolism in sweet potato tuberous roots (TRs) by pot experiment. Compared to the control, drought stress increased soluble sugar and sucrose content by 4–60% and 9–75%, respectively, but reduced starch accumulation by 30–66% through decreasing the starch accumulate rate in TRs. In the drought-treated TRs, the inhibition of sucrose decomposition was attributed to the reduced activities of acid invertase (AI) and alkaline invertase (AKI) and the *IbA-INV3* expression, rather than sucrose synthase (SuSy), consequently leading to the increased sucrose content in TRs. In addition, starch synthesis was inhibited mainly by reducing ADP-glucose pyrophosphorylase (AGPase), granular starch synthase (GBSS) and starch branching enzyme (SBE) activities in TRs under drought stress, and AGPase was the rate-limiting enzyme. Furthermore, soil drought remarkably up-regulated the *IbSWEET11*, *IbSWEET605*, and *IbSUT4* expressions in Jishu 26 TRs, while it down-regulated or had no significant differences in Xushu 32 and Ningzishu 1 TRs. These results suggested that the sucrose-loading capability in Jishu 26 TRs were stronger than that in Xushu 32 and Ningzishu 1 TRs. Moreover, *IbA-INV3*, *IbAGPS1*, *IbAGPS2*, *IbGBSSI* and *IbSBEII* play important roles in different drought-tolerant cultivars under drought stress.

## 1. Introduction

At present, more than a third of the world’s arable land faces water shortages. Drought stress has become one of the main environmental factors limiting crop yields in world under the double exacerbation of the greenhouse effect and climate change [1,2]. How to regulate crop root growth under drought stress is becoming a primary scientific problem [3]. As a typical tuberous root (TR) crop, the sweet potato is a very important food and vegetable crop, industrial raw material and energy crop in China. It has high and stable TR yield, strong resistance to abiotic stress and wide adaptability [4]; however, TR growth and development are susceptible to drought [5]. Therefore, in view of climate change and the development trend of sweet potato production in China, it is necessary to strengthen the research on the physiological ecology of sweet potato TR under drought stress, and to explore regulation methods to avoid drought stress injury in sweet potato production. These results could provide important theoretical values and practical significances to solve low TR yield and poor quality caused by drought stress in sweet potato production. As the main storage carbon, starch accounts for about 50–80% of sweet potato TR’s total mass, and its metabolism is closely related to TR expansion and development process [6,7]. Previous reports have shown that drought stress observably increased sucrose content, and reduced the content of fructose and starch in TRs of potato and sweet potato, which led to a lower number of effective tubers per plant and TR yield [8,9]. These results indicated that, when tuber crops suffered drought, the conversion of sucrose to starch was affected.

Sucrose is the main photosynthate for phloem transport in higher plants. Sucrose transport in plant phloem mainly relies on the involvement of sugar transport genes, such as sucrose transporter (*SUTs*) and sucrose-will-eventually-be-exported transporter genes (*SWEETs*) [10,11]. Drought is one of the main environmental factors that intervenes with the partitioning of carbon through altering photosynthesis, xylem and phloem transport, as well as sugar metabolism [11]. In tuber crops, for sucrose decomposition, acid invertase (Inv) and sucrose synthase (SuSy) have been shown to play key roles in the developing tuber, among which Inv was dominant during the early stage of tuberization, and SuSy predominated at later stages [12]. Yang et al. (2020) found that under drought stress, the overexpression of invertase inhibitor (*IbINH*) in sweet potato increased sucrose content and decreased hexose content and Inv activity, but the opposite results were observed in the down-regulated *IbINH* plants by RNAi technology [13]. In other words, under drought stress, Inv activity and its gene expression in roots were inhibited, and then it affected sucrose decomposition [14]. Other studies found that [15], under water stress, SuSy was the dominant catalyzing enzyme for sucrose decomposition in rice and potato roots, rather than Inv, and its gene expression level and activity were increased. This may be because sucrose decomposition by SuSy had more advantages under water stress than by Inv. Drought stress significantly reduced SuSy activity and the expression levels of its three isozyme genes in cotton anthers [16], which affected sucrose hydrolysis. At present, 13 isoform genes of SuSy have been detected in sweet potato, and most of isoform genes were highly expressed in TRs. Thus, how these isotypes change under drought stress, which isotype is dominant and whether the trend of the isotype gene is consistent with SuSy activity have not been reported, and need further investigation.

In roots of higher plants, starch biosynthesis is mainly catalyzed by ADP-glucose pyrophosphorylase (AGPase), granular starch synthase (GBSS) and soluble starch synthase (SSS); starch branching enzyme (SBE) and debranching enzyme (DBE) are also involved in amylose and amylopectin synthesis and affect starch’s fine structure [17,18,19]. High AGPase, SuSy and SSS activities are conducive to starch synthesis and high yield [7], and the activity of AGPase had a marked positive relationship with the rate of starch accumulation [19]. In rice and triticale grains, moderate drought stress improved the activities of SSS and GBSS, and then increase starch accumulation, but only the amylose content was significantly affected [20]. Under severe drought, in crop sink organs such as wheat and rice grains, and potato tuber the down-regulations of *AGPase*, *SSS* and *GBSS* gene expression and their low activities have led to the restriction of starch synthesis [8,21,22]. Some other reports have showed that the reduced starch content in drought-stressed cassava stems and wheat grains was mainly related to starch decomposition, because the activities of AGPase, GBSS and SSS were improved [23]. Therefore, the mechanism of starch metabolism in drought-stressed crops is different for different crops. The conversion of sucrose to starch had been reported to be catalyzed by multiple isoforms of the enzymes or proteins involved, such as *IbSUT3* and *IbSUT4* (a homologue of *AtSUC2* and *AtSUC4*); *IbSWEET11* (a homologue of *AtSWEET12-like*) and *IbSWEET605* (a homologue of *AtSUC4*); *SuSy2*, *SuSy3* and *SuSy4*; AGPase small subunits 1 and 2 (*AGPS1* and *AGPS2*) [7,24]; *SSSI*, *SSSII*, *SSSIII* and *SSSIV* [25]; *GBSSIa*, *GBSSIb* and *GBSSII* [26]; and *SBEI*, *SBEIIa* and *SBEIIb* [27,28]. Each isoform can have a particular role in the converting process from sucrose to starch [29].

Although many studies have been performed on the relationship between sweet potato TR yield/biomass and photosynthesis, or root morphology or N metabolism under drought stress [2,9,30,31,32,33,34], little is reported about how long-term soil drought affects starch accumulation, according to the enzyme activities and gene expressions in sucrose-starch conversion of sweet potato TRs. Therefore, we speculated that (1) long-term soil drought could inhibit sucrose decomposition through affecting SuSy and invertase activities, (2) affect enzymes and genes related to starch synthesis and (3) sucrose transport capacity could be different among drought-tolerant cultivars.

In this study, we want to (1) investigate the changes of SuSy, AGPase, SSS, GBSS, SBE and DBE and their gene expression levels in sweet potato TRs under long-term soil drought stress, and the relationship with starch accumulation; (2) determine the sucrose-cleaving enzyme, invertase, for changes in its activity and its gene expressions, *IbA-INV2* and *IbA-INV3*; and (3) to clarify the difference among Xushu 32, Ningzishu 1 and Jishu 26 in sucrose transport capacity in response to drought stress.

## 2. Results

### 2.1. Effects of Long-Term Soil Drought Stress on Carbohydrate Content in Sweet Potato TRs

#### 2.1.1. Soluble Sugar and Sucrose Content in TRs

**Figure 1:**
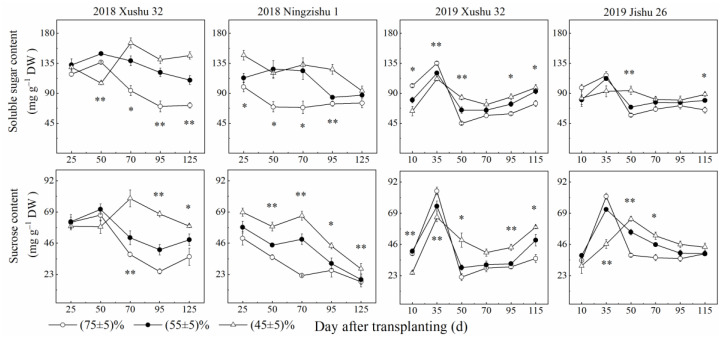
Sugar contents in sweet potato TRs under long-term soil drought stress in 2018 and 2019. * means significant at *p* < 0.05 among the three *SRWC* levels; ** means significant at *p* < 0.01 among the three *SRWC* levels.

The soluble sugar content and sucrose content in sweet potato TRs reduced or first improved and then decreased with increasing DAT for the three *SRWC* levels, and long-term soil drought stress raised soluble sugar content and sucrose content (Figure 1). Compared with the control (*SRWC* (75 ± 5)%), the soluble sugar content and sucrose content in Ningzishu 1 TRs were improved by 38–60% and 33–75%, and by 4–9% and 9–10% in Jishu 26 TRs, under long-term soil drought treatment (*SRWC* (55 ± 5)% and *SRWC* (45 ± 5)%), respectively. For Xushu 32 TRs, the soluble sugar content and sucrose content were raised by 33–40% and 20–42% in 2018, and by 5–9% and 22–66% in 2019, respectively.

#### 2.1.2. Starch Content and Starch Accumulation in TRs

**Figure 2:**
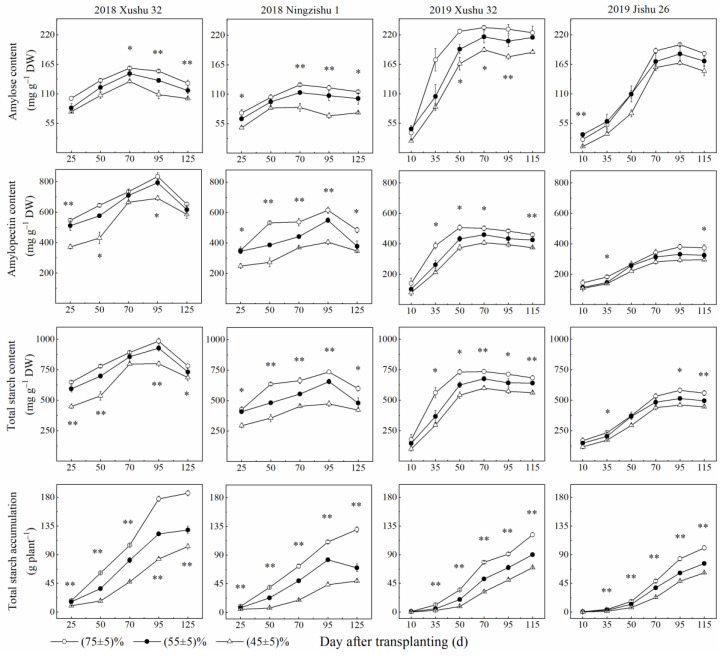
Changes of starch content (amylose and amylopectin) and starch accumulation in TRs under long-term soil drought stress in 2018 and 2019. * means significant at *p* < 0.05 among the three *SRWC* levels; ** means significant at *p* < 0.01 among the three *SRWC* levels.

The contents of amylose and amylopectin in TRs showed an increasing trend with increasing DAT, as well as the total starch content, and were significantly reduced with the *SRWC* decreasing (Figure 2), as well as starch accumulation. In 2018, compared with the control, long-term soil drought stress significantly decreased the contents of amylopectin, amylopectin and total starch in Xushu 32 and Ningzishu 1 TRs by 11–22%; 11–33% and 6–20%; and 17–35%, 7–20% and 16–35%, respectively. These were lower than total starch accumulation (30–52% and 36–66%). In 2019, the changes of these four indicators in Xushu 32 and Jishu 26 TRs were similar with those in 2018, and the decreased amplitude for Xushu 32 was greater than that for Jishu 26.

The starch dynamic accumulation in TRs with increasing DAT were simulated using Logistics Formulas (1)–(3) (Table 1). Compared with the control, the average rate of starch rapid accumulation (*V_T_*) was decreased, as well as the maximum rate (*V_max_*), in the *SRWC* (45 ± 5)%-treated TRs for all three cultivars. However, *SRWC* (45 ± 5)% treatment prolonged the starch rapid accumulation period in Xushu 32 and Ningzishu 1 TRs by 6 d and 14 d, respectively, averaged over two years. Moreover, the coefficient of variation of *V_T_* was larger than that of *T* and *T_m_*.

### 2.2. Effects of Long-Term Soil Drought Stress on Activities of Enzymes in Sucrose-Starch Conversion in Sweet Potato TRs

**Figure 3:**
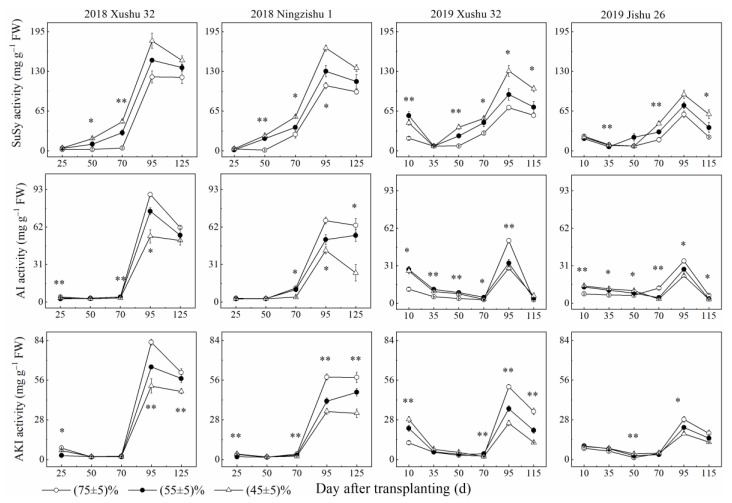
Changes of SuSy, AI and AKI activities in sweet potato TRs under long-term soil drought stress in 2018 and 2019. * means significant at *p* < 0.05 among the three *SRWC* levels; ** means significant at *p* < 0.01 among the three *SRWC* levels.

With increasing DAT, the activities of SuSy, AI and alkaline invertase (AKI) in TRs showed a single peak curve (Figure 3). SuSy activity in TRs was increased with the *SRWC* decreasing. Compared to the control, long-term drought stress increased SuSy activity in TRs of Xushu 32, Ningzishu 1 and Jishu 26 by 43–77%, 30–65% and 36–67%, respectively, averaged over two years. Nevertheless, the activity of AKI and AI in TRs declined with the decrease in *SRWC*.

**Figure 4:**
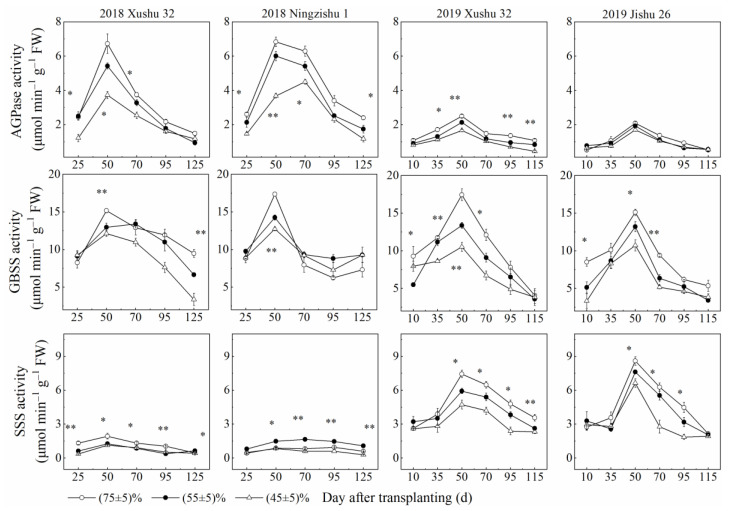
Changes of AGPase, GBSS and SSS activities in sweet potato TRs under long-term soil drought stress in 2018 and 2019. * means significant at *p* < 0.05 among the three *SRWC* levels; ** means significant at *p* < 0.01 among the three *SRWC* levels.

**Figure 5:**
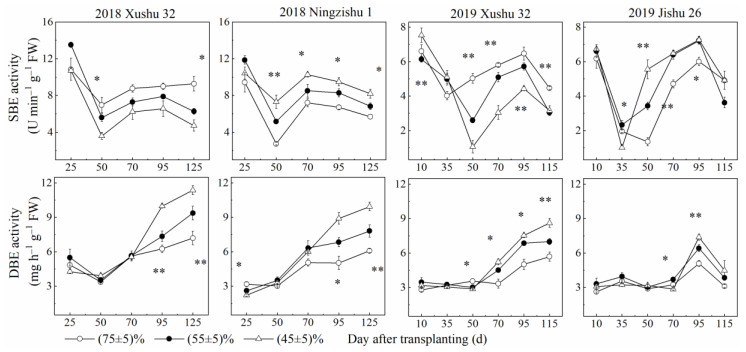
Changes of SBE and DBE activities in sweet potato TRs under long-term soil drought stress in 2018 and 2019. * means significant at *p* < 0.05 among the three *SRWC* levels; ** means significant at *p* < 0.01 among the three *SRWC* levels.

The activities of AGPase, GBSS and SSS in TRs firstly increased and then decreased with increasing DAT. Compared with the control, the activities of the three key enzymes declined with the *SRWC* decreasing (Figure 4), while the activities of SBE and DBE in TRs for the three cultivars were significantly increased, except for SBE activity in Xushu 32 TRs (Figure 5).

### 2.3. Effects of Long-Term Soil Drought Stress on the Genes Expression Levels in Sucrose-Starch Conversion of TRs

**Figure 6:**
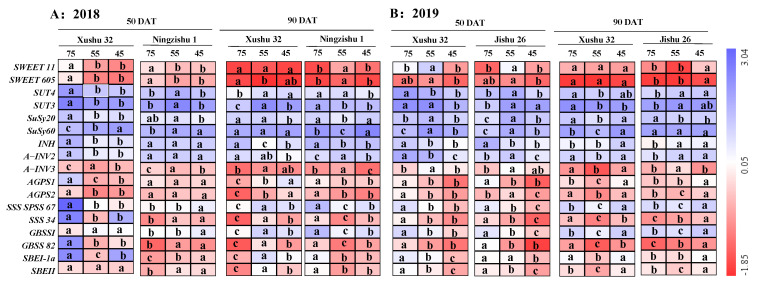
Heat map of the genes’ expression levels in sucrose-starch conversion in TRs. Purple–blue represents increased expression levels, and red represents reduced expression levels. Values followed by different lowercases among the three *SRWC* levels are markedly different at *p* < 0.05 levels.

In 2018 (Figure 6), under long-term soil drought stress (*SWRC* (45 ± 5)%) at 90 DAT, the expression levels of *IbSUT4* were obviously up-regulated by 88% for Xushu 32, but down-regulated by 36% for Ningzishu 1; in 2019, the expression levels of *IbSUT4* were observably increased by 69% for Jishu 26, but there were no significant differences between the control and *SWRC* (45 ± 5)% treatment in *IbSUT4* for Xushu 32. However, the expression level of *IbSUT3* for Jishu 26 was better than for Xushu 32 and Ningzishu 1 at 50 DAT. Under *SWRC* (45 ± 5)% treatment, the expression levels of *IbSWEET11* and *IbSWEET605* had increased by 2.7- and 0.4-fold at 90 DAT for Jishu 26; however, there were no significant differences between the control and *SWRC* (45 ± 5)% treatment in *IbSWEET11* and *IbSWEET605* for Xushu 32.

Moreover, the related genes in sucrose and starch metabolism were also investigated (Figure 6). Compared to the control, long-term soil drought stress obviously decreased the expression levels of *IbSuSy20*, *IbINH* and *IbA-INV2* in TRs at 50 DAT, but increased the expression level of *IbSuSy60* in TRs, with average increases of (0.6–2.9)-fold. In addition, *SWRC* (45 ± 5)% treatment reduced the expression level of *IbA-INV3* by 48% in Ningzishu 1 TRs at 90 DAT, but it had no obvious effect on *IbA-INV3* expression in Xushu 32 and Jishu 26 TRs between the control and *SWRC* (45 ± 5)% treatment. In the drought-treated TRs, the expression levels of *IbAGPS1*, *IbAGPS2*, *IbSSS SPSS67*, *IbGBSSI* and *IbSBEII* were significantly down-regulated at 50 DAT for Xushu 32 and Jishu 26, while the expression levels of these genes were up-regulated for Ningzishu 1. Nevertheless, at 90 DAT, these genes for the three cultivars had an opposite expression trend.

### 2.4. Correlation Analysis of between Starch Accumulation with Its Related Enzymes

Starch accumulation in TRs had significantly positive relationships with *V_T_* and *V_max_*, as well as Al, AKI, AGPase, GBSS and SBE (Table 2). However, it had no obvious differences in starch accumulation with SuSy, SSS and DBE.

## 3. Discussion

Sucrose is the main photosynthate for phloem transport in higher plants, and also the primary source for starch synthesis. Sucrose transport in plant phloem mainly relies on the involvement of sugar transport genes, such as *SWEETs* and *SUTs* [10,11]. In this study, at 50 DAT, drought stress significantly decreased the expressions of *IbSWEET11*, *IbSWEET605*, *IbSUT3* and *IbSUT4* in Xushu 32 TRs (Figure 6), which suggested that the sucrose-loading capacity in TRs were reduced under soil drought stress. Previous studies showed that drought down-regulated the expression level of *SUT1* in cotton pistil, leading to lower pistil carbon accumulation, which was reflected in the declined starch accumulation [35], in accordance with our study. At 90 DAT, under soil drought conditions, the expression levels of *IbSWEET11*, *IbSWEET605* and *IbSUT4* were obviously up-regulated for Jishu 26, while were still down-regulated or had no significant differences for Xushu 32 and Ningzishu 1, compared to the control. These results suggested that the sucrose-loading capacity in Jishu 26 TRs were stronger than that in Xushu 32 and Ningzishu 1 in terms of previous results, which showed that *SUC2* and *SWEET11/12* under drought stress were up-regulated in roots of *Arabidopsis thaliana* and soybean (*Glycine max* L.) [10,11]. It was verified by the results that the increase in soluble sugar and sucrose for Xushu 32 and Ningzishu 1 were greater than that for Jishu 26 (Figure 1).

Sucrose metabolism is also a major factor in TR development under drought stress [9]. In this study, long-term soil drought stress increased SuSy activity and the expression levels of *IbSuSy60* in TRs, but decreased AI and AKI activities and the expression levels of *IbINV2* (Figure 3 and Figure 6). Although the increase in SuSy activity was greater than the decrease in AI activity, the sucrose content in the drought-stressed TRs was still raised, compared to the control. There are two possible causes. One is the increased sucrose phosphate synthase activity [10], the other surmise is that AI plays a dominant role in the later TR development under drought stress. Meanwhile, it was observed that starch accumulation was significantly positively correlated with AI and AKI in TRs, but not significantly correlated with SuSy (Table 2). These results suggested that AI and AKI had more advantages in sucrose decomposition than SuSy, leading to the increased sucrose content in TRs under soil drought stress (Figure 1), which was different from the results of cotton leaf under waterlogging stress [36].

Starch is the main storage form of photosynthates, and is accumulated and stored in sweet potato TRs, later providing energy and carbon for TR development under optimum growth conditions [6,7]. However, in this study, the starch (the sum of amylose and amylopectin) deposition in drought-stressed TRs was inhibited for the three cultivars (Figure 2), consistent with the results of wheat [20]. These results indicated that the demands of energy and carbon during TR development were not met, and finally, the starch accumulation was also reduced in the drought-stressed plants with low *V_T_* and *V_max_* (Figure 2, Table 2). In roots of higher plants, starch biosynthesis is mainly catalyzed by AGPase, GBSS and SSS; SBE and DBE are also involved in the synthesis of amylose and amylopectin and affect the fine structure of starch [17,18,19]. According to this study, compared to the control, long-term soil drought stress down-regulated the expression levels of *IbAGPS1* and *IbAGPS2* and reduced the AGPase activity for the three cultivars (Figure 4 and Figure 6), consistent with the results of a previous study [22]. As a result, long-term soil drought stress inhibited ADP-glucose synthesis as the precursor substrate for amylose and amylopectin formation. Subsequently, for drought-stressed TRs of Xushu 32 and Jishu 26, even the expression levels of *IbSSS SPSS67*, *IbGBSSI* and *IbSBEII* were increased at 90 DAT, and the reduced GBSS, SSS and SBE activities were still observed (Figure 4 and Figure 6), which in turn led to lower amylose and amylopectin synthesis (Figure 2). For drought-stressed TRs of Ningzishu 1, all the expression levels of genes encoded by these enzymes were decreased, leading to a greater decrease in starch content than that for TRs of Xushu 32 and Jishu 26. Moreover, AGPase, GBSS and SBE were positively correlated with starch accumulation in TRs, in according to previous studies [37,38]. As a result, it was speculated that the restrictions of AGPase, GBSS and SBE activities and their genes’ expression levels inhibited overall starch biosynthesis, thus leading to the decrease in starch levels in the drought-stressed TRs.

## 4. Materials and Methods

### 4.1. Plant Materials and Growing Conditions

For the long-term soil drought experiment, Xushu 32 and Ningzishu 1 for 2018, and Xushu 32 and Jishu 26 for 2019 were grown in late June and early July in the anti-rain cover at the Sweet Potato Biology Experimental Station of Jiangsu Normal University, Xuzhou, Jiangsu, China (117°11′ E, 34°15′ N). Healthy and uniform sweet potato seedings with five leaves were cut from TRs and transplanted into pots, with one plant per pot.

The soil at the experiment site was sandy, and collected from the 0–30 cm depth layer in the farmers’ fields. A total of 30 kg of soil was filled into the pots (d = 35 cm, h = 55 cm). In 2018 and 2019, the soil contained, respectively, 12.4 and 13.9 g kg^−1^ organic matter, 0.6 and 0.5 g kg^−1^ total nitrogen and 85.7 and 84.6 mg kg^−1^ available potassium, 32.7 and 35.1 mg kg^−1^ available phosphorus. In each year, 4.8 g of N in the form of urea, 1.6 g of P_2_O_5_ in the form of superphosphate and 3.2 g of K_2_O in the form of potassium chlorate were applied to each pot at basal application. The bulk density and field water holding capacity of the soil were 1.42 g cm^2^ and 0.29 g g^−1^, respectively.

Two rows of pots were placed together. Each plot consisted of 36 pots of plants, and the experiment was a randomized complete block design. Each treatment had three replications. Three water treatment were designed as follows: (75 ± 5)% of soil relative water content (*SRWC* (75 ± 5)%, well-watered, as the control), *SRWC* (55 ± 5)% (mild drought) and *SRWC* (45 ± 5)% (severe drought). After the slow seedling is over (about 7–10 days), soil water treatments were set until the end of harvest stage. Sweet potato plants were watered well before the set of the soil water treatments. Soil water content was determined about every 2–4 days at around 18:00, using a soil sampler to collect the soil from 0–25 cm depth in 2018. Three pots were sampled per plot, and the sample holes were filled with soil from empty pots. The soil samples were measured for the fresh weight, and then were dried at 105 °C in an oven for 8 h. The next day, sweet potato plants would be watered to the upper limit of the appropriate soil moisture limit. In 2019, the soil relative water content was measured by TDR (AZS-100 Handheld Meter), and the soil relative volume content was recalculated and converted to *SRWC*.

### 4.2. Sweet Potato Sampling

Five sweet potato roots or TRs were selected and sampled at 25, 50, 70, 95 and 125 days after transplanting (DAT) in 2018, and at 10, 35, 50, 70, 95 and 115 DAT in 2019 from each treatment. The fresh TRs were washed with ddH_2_O and cut into shreds from the middle of TRs. Some were frozen using liquid nitrogen and stored in a −80 °C refrigerator to measure the enzyme activities and genes levels. The remainder was oven-dried for carbohydrate analysis.

### 4.3. Extraction and Activity Assays for Sugars and Sucrose-Starch Metabolizing Enzymes

Soluble sugar, sucrose and starch (amylose and amylopectin) were determined according to the methods of our laboratory’s previous studies [39,40]. Soluble sugars were extracted from dried TR tissues (0.1 g) with 80% ethanol. Soluble sugar and sucrose in the resuspended supernatant were assayed in term of previously described protocols [39,40]. The amylose and amylopectin were determined according to the dual-wavelength colorimetric method with iodine solution and a microplate reader [28,40].

The sucrose-starch metabolizing enzymes were determined using the methods of our laboratory’s previous studies [39,40]. In details, the frozen sample (0.1 g) in TRs was grinded using liquid N_2_, following an added cool-extraction buffer. The supernatant after centrifugation was used to analyze SuSy, AI, AKI, AGPase, SSS, SBE and DBE activities. In addition, the extraction buffer was added to the precipitate for GBSS analysis. The SuSy, AI, AKI, AGPase, DBE and SBE activities were measured as previous described methods [41,42,43,44]. GBSS activity was assayed by the same method as AGPase.

### 4.4. QRT-PCR Analysis in TRs

Total RNA from the TRs at 50 DAT and 95 DAT were extracted using RNA plant kits (Tiangen, DP441-50T), and then reverse transcribed, and the synthetic cDNA was used for qPCR. In this study, each qPCR result had three biological replications. The primers in this study were shown in Supplementary Table S1. The reference gene was *GAPDH*, and the relative expression levels related in sucrose-starch metabolism were analyzed using the 2^−ΔΔCT^ method [45,46].

### 4.5. Statistical Analysis and Weather Data

Starch dynamic accumulation in TRs is simulated by logistic model:

(1) $$S=\frac{S_{max}}{1+ae^{-b\times DAT}}$$

where *S* (g per plant) is starch accumulation in TRs, *DAT* (d) is days after transplanting, *S_max_* (g) is the maximum accumulation, and *a* and *b* are parameters.

From Formula (1):

(2) $$DAT_{0}=\frac{a}{b},\;DAT_{1}=-\frac{1}{b}ln\frac{2+\sqrt{3}}{a},\;DAT_{2}=-\frac{1}{b}ln\frac{2-\sqrt{3}}{a},\;DAT_{m}=\frac{lna}{b}$$

The maximum rate of starch accumulation in TRs was when *DAT* = *DAT*_0_:

(3) $$V_{max}=\frac{b\times S_{max}}{4},\;V_{T}=\frac{S_{2}-S_{1}}{DAT_{2}-DAT_{1}}$$

where *DAT*_1_ is the days after transplanting when the rapid increase in starch accumulation was started, *DAT*_2_ is the days after transplanting when the rapid increase in starch accumulation was terminated, *DAT_m_* is the days after transplanting when the maximum rate of starch accumulation was reached, *T*(d) = *DAT*_2_ − *DAT*_1_ is the rapid starch increase period, *V_T_* and *V_max_* are the average and maximal rates of starch accumulation.

**Figure 7:**
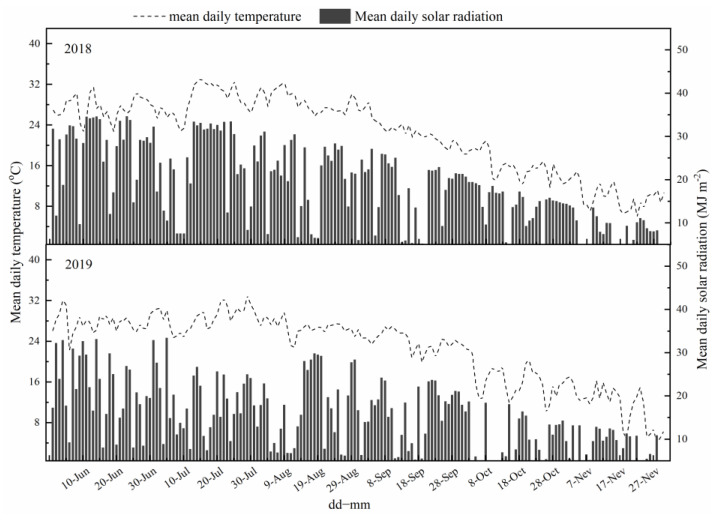
Daily weather in Xuzhou during sweet potato growing period in 2018 and 2019.

Weather data in Xuzhou were collected from the National Metaoralogical Information Center. The average temperature and solar radiation from June to October in 2018 and 2019 were shown in Figure 7. In general, the average solar radiation ware higher in 2018 than in 2019.

The data were recorded and analyzed using Microsoft Excel 2019, Origin 2018 and SPSS 23.0. One-way analysis of variance (ANOVA) was conducted followed by Tukey HSD test at 0.05 and 0.01 levels to examine the differences of measurements under different water treatments within each cultivar. In addition, the statistical analysis of the results was separate for each year.

## 5. Conclusions

Although long-term soil drought stress reduces tuber yield by carbon metabolism in sweet potato TRs, little is reported on how it influences starch metabolism process in TRs. Our results showed that long-term soil drought stress inhibited starch accumulation in TRs by a reduced average and maximum rate. In the drought-treated TRs, the restriction of sucrose decomposition was attributed to the declined activities of AI and AKI and *IbA-INV3* expression, rather than SuSy, consequently leading to the increased sucrose content in TRs. In addition, starch synthesis was inhibited primarily by reducing the activities of AGPase, GBSS and SBE in TRs under long-term soil drought stress, and AGPase was the limiting-rate enzyme in starch synthesis in TRs. Furthermore, the capacity of sucrose loading in Jishu 26 TRs were stronger than that in Xushu 32 and Ningzishu 1 TRs through the up-regulation of *IbSWEET11*, *IbSWEET605* and *IbSUT4* under long-term drought stress, and *IbA-INV3*, *IbAGPS1*, *IbAGPS2*, *IbGBSSI* and *IbSBEII* play important roles in different drought-tolerant cultivars under long-term soil drought stress. In conclusion, the efficient sucrose transport, higher starch accumulation average and maximum rates in TRs may be important functional traits for improving drought tolerance.

## Figures and Tables

**Table 1:** Changes of the eigenvalues of starch accumulation in TRs under long-term soil drought stress in 2018 and 2019.

Year	Cultivar	*SRWC* (%)	R^2^	*S_max_*	t1	T	*V_T_*	*V_max_*
2018	Xushu 32	75 ± 5	0.9901 **	193.0	42.1	31.8	2.4	2.7
		55 ± 5	0.9659 **	155.7	45.2	37.6	1.7	1.8
		45 ± 5	0.9761 **	124.1	51.9	39.9	1.3	1.4
	Ningzishu 1	75 ± 5	0.9949 **	125.7	42.2	27.4	1.8	2.0
		55 ± 5	0.9867 **	96.3	46.8	30.8	1.3	1.4
		45 ± 5	0.9523 **	61.6	58.0	41.0	0.6	0.7
2019	Xushu 32	75 ± 5	0.9847 **	102.5	43.5	18.6	2.2	2.5
		55 ± 5	0.9936 **	88.5	50.2	20.4	1.7	1.9
		45 ± 5	0.9927 **	70.3	57.7	21.1	1.3	1.5
	Jishu 26	75 ± 5	0.9970 **	97.4	55.2	20.4	1.9	2.1
		55 ± 5	0.9898 **	76.8	56.8	21.7	1.4	1.6
		45 ± 5	0.9865 **	61.8	61.0	19.2	1.3	1.4

*S_max_*, maximum starch accumulation; t1, start time of the starch rapid-accumulation period; T, starch rapid accumulation period; *V_max_*, maximum rate of starch accumulation; *V_T_*, average rate of starch accumulation. ** means significant at *p* < 0.01.

**Table 2:** Correlation analysis between starch accumulation with *T*, *V_T_*, *V_max_*, and sucrose-starch metabolizing enzymes.

Correlation with	*T*	*V_T_*	*V_max_*	SuSy	AI	AKI	AGPase	GBSS	SSS	SBE	DBE
Starch accumulation	0.391	0.662 *	0.664 *	0.128	0.828 **	0.894 **	0.578 *	0.741 **	−0.396	0.599 *	0.419

*T* means the fast starch accumulation phase; *V_T_* means the average rate during the fast starch accumulation phase; *V_max_* means the maximum accumulation rate; SuSy means sucrose synthase; AI means acid invertase; AKI means alkaline invertase; AGPase means ADP-glucose pyrophosphorylase; GBSS means granule-bound-starch synthase; SSS means soluble starch synthase; SBE means starch-branching enzyme; DBE means starch-debranching enzyme. n = 12; * means significant at *p* < 0.05; ** means significant at *p* < 0.01. R_0.05_ = 0.5760, R_0.01_ = 0.7079.

## Data Availability

Not applicable.

## Supplementary Materials

The following are available online at https://www.mdpi.com/article/10.3390/ijms24033053/s1.
