# Supplementary material for: Long-Term Soil Drought Limits Starch Accumulation by Altering Sucrose Transport and Starch Synthesis in Sweet Potato Tuberous Root
Source: Int J Mol Sci. 2023 Feb 3;24(3):3053. doi: 10.3390/ijms24033053 (PMC9918156; doi:10.3390/ijms24033053)
Supplement: Supplementary file 1 [file ijms-24-03053-s001.zip › ijms-2158269-supplementary.pdf]

Table S1. Specific primers for gene amplification.

Target gene	Forward primer（5'—3'）	Reverse primer（5'—3'）	Gene ID
GAPDH	ATACTGTGCACGGACAATGG	TCAGCCCATGGAATCTCTTC	JX177362.1
Sweet11	GCTGAGCTGACAGAGGAACA	CTTGGACGATTCAGGTCCCC	NM_114733.4
Sweet605	TGGTGCACCCTGTGGATTCAGGGA	TCACTTGATGGACTCAGCCGGCCA	comp82605_c0_seq1_
SUT4	AGCTTCGACGAGCAGAGAAC	CCCATCCGAACTGTATCCCG	MN233360.1
SUT3	GCGACGCCGATATGGAAGAT	GTAAGGGGTGAGGAGGGAGA	MN233361.1
SuSy20	CTTGAGATTCGTCGCTACCTT	CTGAACCCTCCCTTCATCTTAC	EU908020.1
SuSy60	TTCATCCACTTTCCTTCTTCATCTA	TGCTCTCAAATACCGCAAGT	CA409460.2
INH	TCCTCTGTCCTAAATGGGGGAA	GTAGTTGGGGGTGTTGTTGC	AF529166.1
A-INV2	TGGCTTCCGGGGTTGAAAAG	TGATCACCGTTCTTCCTCCT	AY037937.1
A-INV3	CACTCGATCGTAGAGAGCTTTG	ATCTTCACCGACGCCTTCAC	AY037938.2
AGPS1	GCAGACTTGTCTAGATCCTGATG	CCGCTTCTTTGTGAGAGGATAG	JQ797696.1
AGPS2	AACTCGGCTCGTTAGTTTCTC	GGGAGTCGGAAACAGCTTTA	JQ797697.1
SSS SPSS67	GGAATGGCAAGCAGGAACTA	CCTCCAAGAACATCTCCAAGTC	U44126.1
SSS34	GACTGTGGGATCTACTGAAAGG	TTGCTGGCTCCTGAGAATTTA	AF068834.1
GBSSI	GGAATGGCAGCAGGAACTAT	CATCTCCAAGTCCACCAGTTT	AB524727.1
GBSS82	TGGCATGTTCCGTGCGGTGG	CCCGCGACCCTGGTGAGCTA	comp82416_c3_seq5
SBEI-1a	CCTTCTCGTGGGTCTTTCATAC	ACCAGTAGTGCATGGTGAAG	AB194722.1
SBEII	CCTATGAATCCGAACCCTCTTC	ATGCCTCAGCAACCTCTAAC	AB071286.1
